# Mesiodistal width correlation between primary and successor mandibular teeth: implication for early orthodontic diagnosis

**DOI:** 10.3389/fdmed.2025.1659242

**Published:** 2025-09-24

**Authors:** Aleyna Cakir, Annika Both, Christian Kirschneck, Nikolaos Daratsianos, Cristiano Miranda de Araújo, Juliane Corá, Erika Calvano Küchler, Svenja Beisel-Memmert

**Affiliations:** ^1^ Department of Orthodontics, Medical Faculty, University Hospital Bonn, Bonn, Germany; ^2^ Department of Orthodontics, Medical Faculty, University of Tuiuti of Paraná, Curitiba, Brazil

**Keywords:** primary teeth, mesiodistal width, tooth size prediction, mandibular dentition, mixed dentition analysis, orthodontic diagnosis

## Abstract

**Background:**

Most studies on permanent tooth width prediction focus on the predictive value of permanent teeth, however only a few studies examine the predictive value of primary teeth. The aim of this study was to investigate the correlation between the mesiodistal widths of the mandibular primary canines and molars and those of their permanent successors. In addition, the study evaluated whether the mesiodistal width of the primary canines and molars can serve as reliable predictors for the width of the permanent mandibular first molars.

**Methods:**

This cross-sectional study analyzed records from 143 orthodontic patients (78 males and 65 females) who had digitized dental models in the mixed and in the permanent dentition stage. Mesiodistal measurements were performed on left-sided mandibular permanent teeth (canines, first and second premolars, first molar), and primary teeth (canines, first and second molars). The Pearson correlation coefficient test was used to determine the correlation strength between the mesiodistal dimensions of primary and permanent teeth (*p* < 0.05).

**Results:**

Significant correlations were found between second primary molars and second premolars (Pearson *r* = 0.400–0.461) as well as between primary and permanent canines (Pearson *r* = 0.462–0.512), across the total sample and within both sexes. The dimensions of all three evaluated primary teeth were correlated with first permanent molar with r ranging from 0.402 to 0.625. The primary first molar showed a weak correlation with the first premolar for the total sample (Pearson *r* = 0.240) and males (Pearson *r* = 0.302), and none was observed for female patients (Pearson *r* = 0.048).

**Conclusions:**

A link between primary and permanent tooth width of canines and posterior dentition was observed, but a difference between sexes exists. Therefore, primary teeth may offer early insight into future space requirements, however their predictive strength is influenced by tooth type and sex.

## 1 Introduction

Tooth development is an integral component of craniofacial growth and development, influencing oral function, aesthetics, and overall health (1). The formation of the primary (deciduous) teeth and permanent teeth begins *in utero* during the first trimester (2) and involves complex genetic, molecular and cellular mechanisms (3). The eruption of primary teeth typically begins around the eighth postnatal month, following a sequential pattern throughout early childhood (2).

Primary teeth play a crucial role in oral health by influencing mastication, supporting verbal development, and ensuring proper positioning of their permanent successors (4). Around the age of six, the gradual transition to permanent dentition begins with the eruption of the first molars, marking the onset of the mixed dentition stage (2). This key phase of craniofacial growth is characterized by complex morphological and structural adaptations within the dental arches and skeletal structures (5). A coordinated sequence of tooth eruption and jaw growth is essential for achieving occlusal and dentofacial harmony (5).

Discrepancies between mesiodistal crown width and available arch space can result in malocclusions, which not only impair oral function and reduce masticatory efficiency but also increase the risk of dental health issues, such as periodontal disease (6, 7). Therefore, the prediction of mesiodistal tooth dimensions is subject of fundamental importance in orthodontics and pediatric dentistry diagnosing potential crowding issues and allowing for early interventions.

Most studies on tooth width prediction have focused on permanent teeth, particularly the incisors (8), due to their high morphological consistency (9). The established methods developed by Moyers and by Tanaka and Johnston rely on the mesiodistal widths of erupted permanent mandibular incisors to estimate the mesiodistal dimensions of unerupted permanent canines and premolars (10, 11). However, only a limited number of studies have examined the predictive value of primary teeth (12–14).

Nuvvula et al. (12) aimed to predict the mesiodistal dimensions of permanent canines and premolars using the Boston University method described by Gianelly and conducted a comparative analysis with the Tanaka–Johnston approach. Their analysis was based on the mesiodistal widths of primary maxillary and mandibular canines and first molars. Statistically significant correlations were found between the predicted values from both methods, particularly among female subjects (12).

In a Spanish population, Bravo et al. (13) identified a statistically significant correlation between the mesiodistal dimensions of primary second molars and permanent first molars. Al-Dulaimy et al. (14) corroborated these findings in an Iraqi cohort and extended them by developing regression equations to estimate permanent first molar width based on the dimensions of primary second molars.

These findings underscore the relevance of primary teeth in mixed dentition analysis and highlight the need for further studies assessing tooth-specific correlations with permanent successors. In particular, the predictive value of primary canines and first molars for estimating permanent first molar widths remains unclear. To our knowledge, no study has investigated the mesiodistal width correlations between mandibular primary canines and molars and both their permanent successors and the permanent first molars within the same cohort.

The current study aimed to investigate the mesiodistal width correlation between mandibular primary canine and molars with permanent successors considering sex-related differences. Therefore, the study had two main objectives: 1) to investigate the mesiodistal width correlation between mandibular primary canine and molars with their permanent successors; and 2) to determine whether primary canine and molars can serve as reliable predictors of permanent mandibular first molar widths.

## 2 Material and methods

### 2.1 Sample collection and inclusion criteria

This study involved digitized dental models of 143 orthodontic patients (78 males, 65 females) aged between 4.7 and 13.6 years (9.34 ± 1.70 years) who were treated in the Orthodontic Department of the University Hospital Bonn (Germany). According to the principles of the Declaration of Helsinki the study was approved by the local Ethics Committee (No. 2024-100-BO). Written informed consent was taken from the patients and parents of patients under 18 years of age who participated in the present study.

Initially, orthodontic records of 1,432 patients were screened for those who had at least two dental plaster models in the mixed dentition stage and the permanent dentition stage. The conventional dental casts were digitized using the Scanner S600 ARTI (Zirkonzahn, Neuler, Germany).

Children with syndromes, oral clefts, or cases of bilaterally missing permanent teeth were not included in the study. Additionally, singular teeth presenting developmental alterations (such as shape or structure anomalies), carious lesions, enamel or dentin fractures, proximal fillings or prosthetic restorations were excluded.

The evaluation was performed by a single trained dentist. For intra-examiner reliability, ten randomly chosen models were evaluated twice in a two-week interval and the reliability was examined using intra-class correlation coefficients (ICC) and their 95% confidence intervals (ICC = 0.99).

The sample size estimation was performed assuming an alpha of 0.05, a beta of 0.20 and a minimum r (expected correlation coefficient) of 0.30, which predicted a minimum sample of 62 using sample-size.net (https://sample-size.net/correlation-sample-size/). Therefore, a minimum sample of 124 patients (62 of each sex) was established.

### 2.2 Mesiodistal measurement

**Figure 1:**
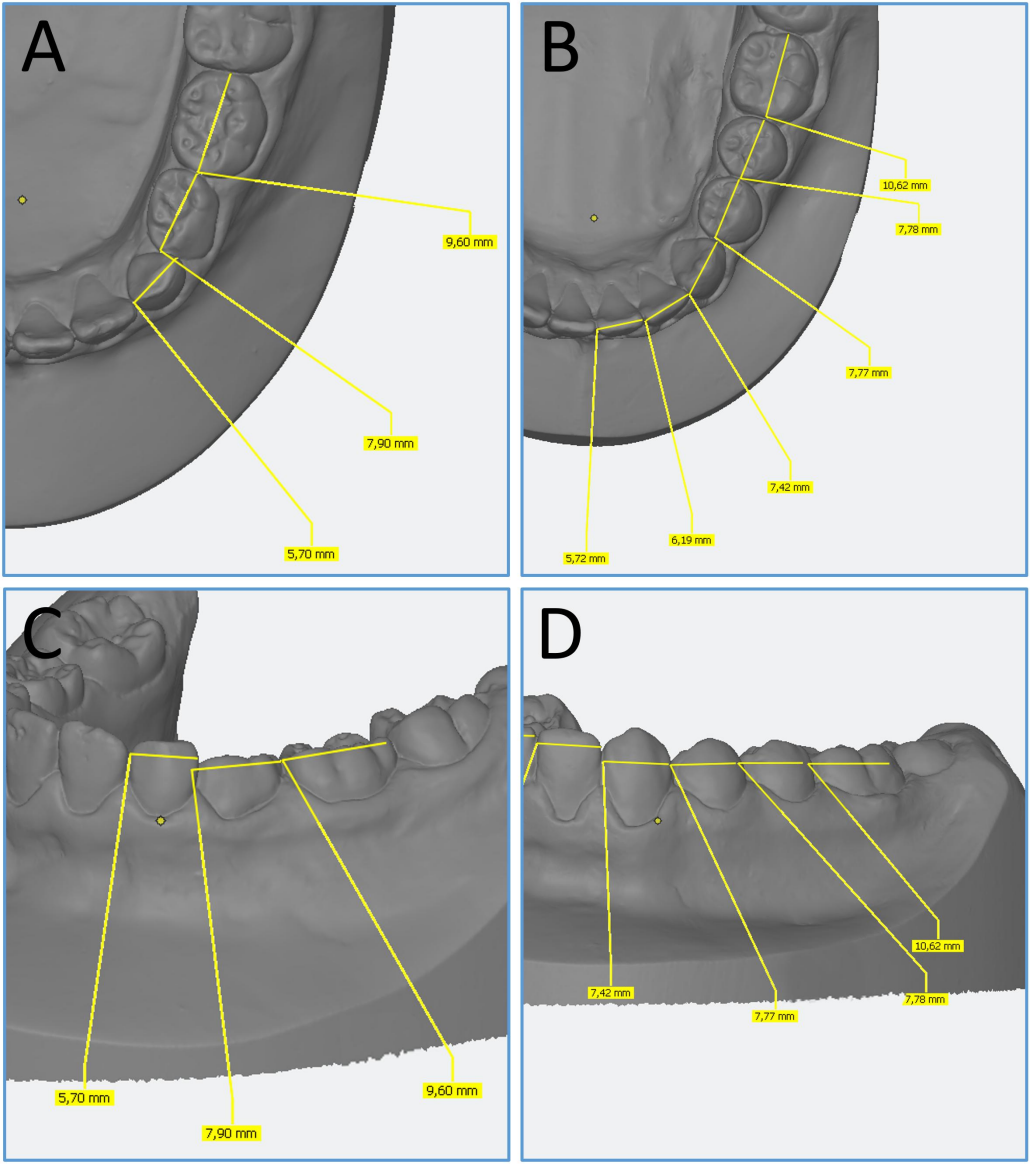
Onyxceph^3^ LAB: mesiodistal measurements of primary and permanent teeth. **(A,B)** Occlusal views of primary **(A)** and permanent teeth **(B)**. **(C,D)** Buccal views of primary **(C)** and permanent teeth **(D)**.

The mesiodistal measurements of dental plaster models were performed with the dental imaging software OnyxCeph^3^™ (Image Instruments, Chemnitz, Germany). The mesiodistal dimension of each tooth was determined as the maximum distance between its interproximal contact points, measured perpendicular to its long axis. Firstly, the teeth were segmented using semi-automatic or manual tools. The most mesial and distal contact points were automatically generated on each tooth defining the maximum horizontal width of the tooth. After the landmarks were set, the mesiodistal dimension was calculated as the linear distance between these two points in millimeters (Figure 1).

The mesiodistal measurements were performed on the left side of the following mandibular permanent canines, first and second premolars, first molar and primary canines, first and second molars. Teeth which did not meet the inclusion criteria (one canine, one first molar, and two second molars) were replaced with the corresponding contralateral teeth.

### 2.3 Statistical analysis

Data were analyzed using the GraphPad Prism statistics software (Version 9.3.1 for Windows, GraphPad Software, San Diego, California, USA). The normal distribution of the data was examined using the Shapiro–Wilk test. The Pearson correlation coefficient test was used to determine the correlation strength between the mesiodistal dimensions of primary and permanent teeth, and the strength of the correlations was determined according to the value of the “Correlation Coefficient”. The analysis was performed and described in the total sample and stratified according to the sex (males and females). T-test was used to compare sex morphometric differences. The adopted alpha established as 5% for all comparisons (*p* < 0.05).

## 3 Results

Firstly, the mean distribution of the mesiodistal width of primary and permanent teeth in the total sample and in relation to sex was analyzed. Our results indicate that males had statistically significant larger permanent teeth than females (t-test; *p* < 0.05). In contrast, a statistical significant difference of mesiodistal dimensions between both sexes was not observed for primary teeth (t-test; *p* > 0.05) (Table 1). First and second primary molars were larger than their permanent successors (first and second premolars), while primary canines were smaller than permanent canines. These data are shown in the Table 2.

**Table 1:** Mesiodistal dimensions of the evaluated teeth and comparisons among sexes.

Tooth type	Total					Males					Females					*p*-value^#^
	*n*	Min (mm)	Max (mm)	Mean	SD	*n*	Min (mm)	Max (mm)	Mean	SD	*n*	Min (mm)	Max (mm)	Mean	SD	
Primary canine	108	4.72	6.61	5.71	0.36	66	4.72	6.61	5.74	0.39	42	4.93	6.45	5.66	0.29	0.2479
Primary first molar	113	6.38	8.58	7.76	0.39	64	6.38	8.58	7.79	0.45	49	7.10	8.30	7.73	0.30	0.4198
Primary second molar	143	8.45	11.03	9.89	0.46	78	8.45	11.03	9.93	0.50	65	8.98	10.70	9.85	0.41	0.2815
Permanent canine	143	5.95	8.10	6.89	0.43	78	6.06	8.10	7.02	0.46	65	5.95	7.64	6.59	0.33	<0.0001*
Permanent first premolar	143	6.04	8.45	7.22	0.43	78	6.22	8.45	7.30	0.46	65	6.04	8.03	7.09	0.38	0.0032*
Permanent second premolar	143	6.14	8.60	7.32	0.47	78	6.23	8.60	7.42	0.47	65	6.14	8.41	7.20	0.45	0.0062*
Permanent first molar	143	9.68	12.83	11.27	0.59	78	9.86	12.83	11.49	0.56	65	9.68	12.03	11.00	0.52	<0.0001*

Min means minimum, max means maximum and SD means standard deviation.

^#^ *t* test was used.

*Means statistical difference.

**Table 2:** The mean difference between primary and permanent teeth (in millimeters).

Tooth type	Mean difference (mm) (95% confidence interval)		
	Total	Males	Females
Primary second molar	−2.58 (−2.68 to −2.47)	−2.61 (−2.76 to −2.46)	−2.62 (−2.78 to −2.46)
Primary first molar	−0.55 (−0.66 to −0.44)	−0.57 (−0.72 to −0.41)	−0.63 (−0.78 to 0.47)
Primary canine	1.18 (1.07 to 1.285)	1.27 (1.13 to 1.42)	0.96 (0.81 to 1.12)

**Figure 2:**
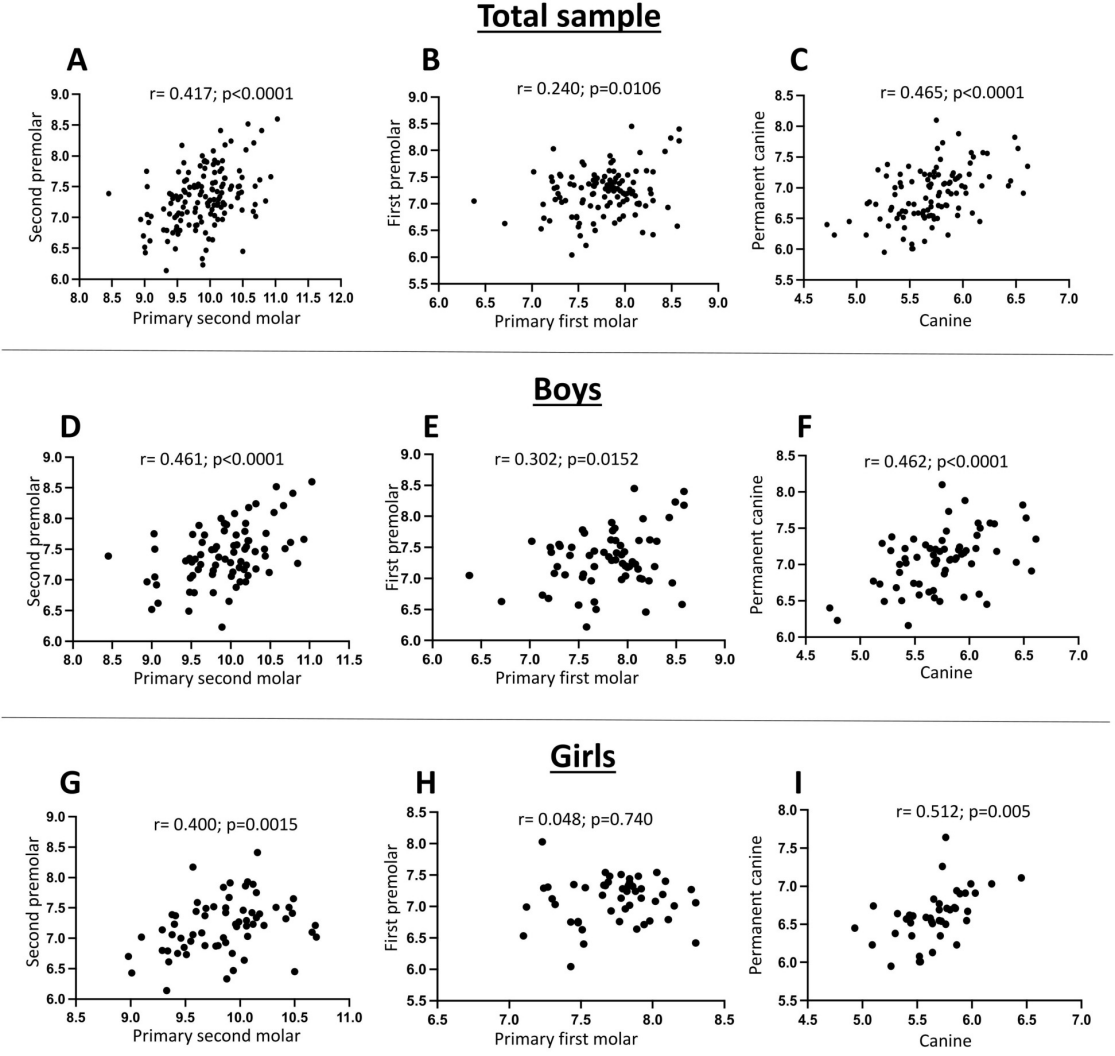
Correlation between the mesiodistal width of primary teeth and their successor permanent teeth. **(A,D,G)** Correlation between primary second molar and permanent second premolar for the total sample **(A)**, males **(D)**, and females **(G)**. **(B,E,H)** Correlation between primary first molar and permanent first premolar for the total sample **(B)**, males **(E)**, and females **(H)**. **(C,F,I)** Correlation between primary canine and permanent canine for the total sample **(C)**, males **(F)**, and females **(I)**.

Secondly, the relationship between primary canine and primary molars with their permanent successors for both sexes (Figures 2A–C), and for male (Figures 2D–F) and female patients (Figures 2G–I) was analyzed separately. A moderate correlation was observed in almost all comparisons. A moderate positive correlation was found between the widths of second primary molars and their permanent successors, as well as between primary and permanent canines, across the total sample and within both sexes groups (Pearson *r* ranging from 0.400 to 0.461 or from 0.462 to 0.512, respectively). The primary first molar showed a weak correlation with the first premolar for the total sample (Pearson *r* = 0.240) and males (Pearson *r* = 0.302), and none was observed for female patients (Pearson *r* = 0.048) (Figure 2).

The analysis between primary canine and molars with permanent first molar revealed that the dimensions of all three evaluated primary teeth (canines, first and second molars) were moderately correlated with first permanent molar, with Pearson r ranging from 0.402 to 0.625 (Table 3).

**Table 3:** The correlation between permanent first molar and primary canines and molars.

Tooth type	Permanent first molar					
	Total		Males		Females	
	*r*	*p*-value	*r*	*p*-value	*r*	*p*-value
Primary second molar	0.547	<0.0001	0.625	<0.0001	0.462	0.0001
Primary first molar	0.468	<0.0001	0.533	<0.0001	0.402	0.0092
Primary canine	0.469	<0.0001	0.430	0.0003	0.408	0.0073

All correlations were statistically significant (*p* < 0.05).

## 4 Discussion

The present study aimed to examine the correlation between the mesiodistal dimensions of primary and permanent canines and the posterior dentition, with particular focus on their potential to predict the mesiodistal width of the permanent teeth and manage the available and required space, taking the sex into consideration. An improved understanding of the relationship between primary and permanent teeth dimension could increase early assessment of potential space deficiencies, enabling timely orthodontic intervention and optimizing treatment planning.

Our analysis shows statistically significant moderate correlations between the mesiodistal widths of primary canines and second molars and those of their permanent successors. These relationships may be attributed to shared developmental pathways, including their origin from the successional lamina and exposure to similar morphogenetic influences during odontogenesis (15). In contrast, primary first molars exhibited non-significant association with the mesiodistal width of the first premolar, particularly in females. This observation may reflect the morphological variability characteristic of primary first molar and potential sex-linked differences in early dental development, which should be take into consideration in the space prediction (16, 17).

In relation to the permanent first molar, primary canines and primary molars showed moderate correlations, with predictive strength varying by tooth type. Consistent with earlier findings, the primary second molar demonstrated the highest correlation, particularly in males (13, 14, 18). This finding is in line with previous studies that reported similar relationships between the mesiodistal dimensions of primary second and permanent first molars, further supporting the predictive relevance of the primary second molar (13, 14, 18).

The primary second molar is considered the closest morphological analogue to the permanent first molar, a natural observation referred to as *isomorphism* (13). Permanent molars are classified as non-successional teeth and develop independently of the successional lamina (15). Since the permanent first molar develops during the continued formation of the primary second molar, this anatomical and temporal overlap may result in similar genetic, hormonal, and growth-related impacts.

Our findings observed the well-established pattern of larger permanent teeth in males, consistent with earlier research across diverse populations (19–21). Zorba et al. (21) observed that sexual dimorphism is generally more pronounced in European populations and among African Americans, Australian Aborigines, and Mexicans, while lower levels were reported in Asian and South American populations. They also noted that canines tend to exhibit the highest degree of dimorphism across most groups. This population-specific variability of sexual dimorphism reflects a complex interaction of multifactorial influences on dental development (21). Alvesalo (22) emphasized the role of sex chromosomes in enamel and dentin development, providing a genetic basis for observed differences in tooth dimensions. In contrast, our study found no statistically significant sex differences in the primary dentition, a result consistent with previous findings (23, 24). As suggested by Lukacs, the reduced dimorphism in primary teeth may be attributed to the limited role of postnatal sex hormones, as primary tooth formation begins during early prenatal development (23). This assumption is further supported by Gil-Donoso et al. (24), who observed no significant sex-related differences in enamel and dentin proportions of primary canines. Primary teeth size appears to be more strongly genetically controlled than permanent teeth, as hormonal effects increase during puberty (24, 25).

The present study provides evidence for the predictive utility of primary canines and molars in estimating the mesiodistal width of the mandibular permanent back teeth. Among all examined tooth types, the primary second molar exhibited the strongest correlation with its successor and with first mandibular molar, indicating a higher degree of reliability within this subgroup. In comparison, the primary first molar demonstrated a weak correlation with its permanent successor for the total sample and for male patients, while no correlation was observed in females. This indicates that while certain primary teeth may offer early insight into future space requirements, their predictive strength is influenced by both tooth type and sex.

The early mixed dentition phase represents a clinically reliable period for evaluating the risk of malocclusion and space deficiency (26). At this stage, space analysis enables the early identification of potential discrepancies, forming the basis for timely orthodontic intervention. In light of the present findings, primary second molars may be considered potential indicators in early treatment planning, particularly with regard to interceptive strategies such as space maintenance or serial extraction (27). Early intervention has been associated with a reduced need for more extensive treatment in adolescence (1, 26). In line with this, the American Academy of Pediatric Dentistry emphasizes the relevance of eruption guidance and growth monitoring as integral components of pediatric oral health care (1). Moreover, early management of dentofacial anomalies during growth contributes not only to improve functional and aesthetic outcomes but also to long-term treatment stability (1).

Advancements in digital technologies have further enhanced the ability to perform early diagnostic measurements with greater precision and reproducibility (5, 19). Intraoral scanners and three-dimensional imaging techniques allow for non-invasive, reproducible measurements and provide a reliable alternative to conventional methods (5, 19).

The longitudinal design of the present study allows for the observation of developmental changes from primary to permanent dentition within the same individuals. This design minimizes inter-individual variability and strengthens the internal validity of the findings. However, the homogeneity of the study sample limits the generalizability of the findings to other populations.

It is also important to mention that the analysis focused on the left mandibular side, which limits the generalizability of the findings to right side. To improve external validity, future studies should aim to replicate these results across distinct populations and evaluating both sides (left and right). Standardized digital measurement methods, combined with population- and sex-specific datasets, may contribute to the development of clinically applicable tools for space analysis and individualized treatment planning in pediatric dentistry to predict the required space in millimeters.

## 5 Conclusion

A correlation between primary and permanent canine and posterior dentition was observed. Our results show that primary canine and molars could be seen as useful diagnostic tool to predict permanent teeth dimension. These primary teeth may offer early insight into future space requirements, however their predictive strength is influenced by both tooth type and the patient's sex.

## Data Availability

The raw data supporting the conclusions of this article will be made available by the authors, without undue reservation.

## References

[1] American Academy of Pediatric Dentistry. Management of the developing dentition and occlusion in pediatric dentistry. In: American Academy of Pediatric Dentistry, editor. The Reference Manual of Pediatric Dentistry. Chicago, USA: American Academy of Pediatric Dentistry (2024). p. 475–93.

[2] Proffit WR. Early stages of development. In: Proffit WR Fields HW Larson BE Sarver DM, editors. Contemporary Orthodontics. 6th ed. Philadelphia, USA: Elsevier (2019). p. 65–77.

[3] Rathee M Jain P. Embryology, teeth. (2023). https://www.ncbi.nlm.nih.gov/books/NBK560515/ (Accessed March 23, 2025).

[4] Lynch RJM. The primary and mixed dentition, post-eruptive enamel maturation and dental caries: a review. Int Dent J. (2013) 63:3–13. 10.1111/idj.12074 24283279 PMC9375027

[5] Koaban A Alotaibi SS Abu Nakha KM Bin Huraib S Alhassan MH Rubayan AR Orthodontic space management in pediatric dentistry: a clinical review. Cureus. (2024) 16:e76026. 10.7759/cureus.76026 39835004 PMC11743602

[6] Joshi N Hamdan AM Fakhouri WD. Skeletal malocclusion: a developmental disorder with a life-long morbidity. J Clin Med Res. (2014) 6:399–408. 10.14740/jocmr1905w 25247012 PMC4169080

[7] Leck R Paul N Rolland S Birnie D. The consequences of living with a severe malocclusion: a review of the literature. J Orthod. (2022) 49:228–39. 10.1177/14653125211042891 34488471 PMC9160782

[8] Abaid S Zafar S Kruger E Tennant M. Size estimation of unerupted canines and premolars using various independent variables: a systematic review. J Orofac Orthop. (2023) 84:164–77. 10.1007/s00056-022-00392-9 35420320

[9] Moorrees CF Reed RB. Correlations among crown diameters of human teeth. Arch Oral Biol. (1964) 9:685–97. 10.1016/0003-9969(64)90080-9 14219519

[10] Tanaka MM Johnston LE. The prediction of the size of unerupted canines and premolars in a contemporary orthodontic population. J Am Dent Assoc. (1974) 88:798–801. 10.14219/jada.archive.1974.0158 4525402

[11] Moyers RE. Handbook of Orthodontics. 4th ed. Chicago, USA: Year Book Medical Publishers (1988).

[12] Nuvvula S Vanjari K Kamatham R Gaddam KR. Primary dentition analysis: exploring a hidden approach. Int J Clin Pediatr Dent. (2016) 9:1–4. 10.5005/jp-journals-10005-1323 27274146 PMC4890053

[13] Bravo N Facal M Maroto M Barbería E. Relationship between mesiodistal crown diameters of permanent first molars and deciduous second molars. Eur J Paediatr Dent. (2010) 11:115–21. 21080750

[14] Al-Dulaimy DA Nahidh M Al-Khannaq MRA. Predicting the mesiodistal crown dimensions of the permanent first molars from the deciduous second molars. ScientificWorldJournal. (2021) 2021:9315553. 10.1155/2021/9315553 34220368 PMC8221863

[15] Nanci A. Development of the tooth and its supporting tissues. In: Nanci A, editor. Ten Cate’s Oral Histology: Development, Structure, and Function. 9th ed. St. Louis, USA: Elsevier (2018). p. 217–19.

[16] Tsai HH. Descriptive classification of variations in primary mandibular first molars. ASDC J Dent Child. (2001) 68:23–6. 11324402

[17] Poornima P Pathak S Nagaveni N Roopa K. Unusual morphology of primary mandibular first molar. Niger J Exp Clin Biosci. (2015) 3:57–8. 10.4103/2348-0149.158170

[18] Hussain T Rasool G Zahra FT Hussain U Bano S. The relation between the mesiodistal crown widths of the deciduous second molars and the permanent first molars. Pakistan Oral Dent J. (2016) 36:71–4.

[19] Abaid S Zafar S Kruger E Tennant M. Mesiodistal dimensions and sexual dimorphism of teeth of contemporary western Australian adolescents. J Oral Sci. (2021) 63:247–51. 10.2334/josnusd.20-0596 34011827

[20] Litha, Girish HC Murgod S Savita JK. Gender determination by odontometric method. J Forensic Dent Sci. (2017) 9:44. 10.4103/jfo.jfds_96_15 PMC5450487 28584479

[21] Zorba E Moraitis K Manolis SK. Sexual dimorphism in permanent teeth of modern Greeks. Forensic Sci Int. (2011) 210:74–81. 10.1016/j.forsciint.2011.02.001 21371836

[22] Alvesalo L. Human sex chromosomes in oral and craniofacial growth. Arch Oral Biol. (2009) 54:S18–24. 10.1016/j.archoralbio.2008.06.004 18657798

[23] Lukacs JR. Sexual dimorphism in deciduous tooth crown size: variability within and between groups. Am J Hum Biol. (2022) 34:1–17. 10.1002/ajhb.23793 36054733

[24] Gil-Donoso E García-Campos C Blasco-Moreno S Modesto-Mata M Martínez De Pinillos M Moreno-Torres C Sexual dimorphism of deciduous canine dental tissues dimensions of modern human populations. Anthropol Sci. (2023) 131:107–15. 10.1537/ase.230315

[25] Garn SM Lewis AB Kerewsky RS. Genetic, nutritional, and maturational correlates of dental development. J Dent Res. (1965) 44:228–42. 10.1177/00220345650440011901 14242327

[26] Gaur S Singh N Singh R Phukan AH Mittal M Kohli A. Mixed dentition analysis in and around Kanpur city: an existential and illustrative study. Int J Clin Pediatr Dent. (2022) 15:603–9. 10.5005/jp-journals-10005-2470 36865713 PMC9973109

[27] Al-Bitar ZB Al-Omari IK Sonbol HN Al-Ahmad HT Hamdan AM. Mixed dentition analysis in a Jordanian population. Angle Orthod. (2008) 78:670–5. 10.2319/0003-3219(2008)078[0670:MDAIAJ]2.0.CO;2 18302466

